# Augmentation of bone formation by sympathectomy in rats as evaluated by [^99m^Tc]Tc-MDP

**DOI:** 10.3389/fendo.2025.1580230

**Published:** 2025-07-02

**Authors:** Zili Cai, Xiuting Lin, Yuehong Zhuang, Weibing Miao, Yun Xie

**Affiliations:** ^1^ Department of Orthopaedic Surgery, National Regional Medical Center, Binhai Campus of the First Affiliated Hospital, Fujian Medical University, Fuzhou, China; ^2^ Department of Orthopaedic Surgery, First Affiliated Hospital, Fujian Medical University, Fuzhou, China; ^3^ Department of Nuclear Medicine, National Regional Medical Center, Binhai Campus of the First Affiliated Hospital, Fujian Medical University, Fuzhou, China; ^4^ Department of Nuclear Medicine, First Affiliated Hospital, Fujian Medical University, Fuzhou, China; ^5^ Fujian Key Laboratory of Brain Aging and Neurodegenerative Diseases, Institute of Clinical Applied Anatomy, Fujian Medical University, Fuzhou, China

**Keywords:** bone formation, sympathectomy, [99m Tc]Tc-MDP, SPECT/CT imaging, rats

## Abstract

**Background:**

The role of the sympathetic nervous system in bone metabolism remains unclear. Given that ^99m^Tc-methylene diphosphonate ([^99m^Tc]Tc-MDP) uptake reflects active bone formation and mineralization, this study aims to investigate the effects of sympathetic denervation on bone formation in rats using [^99m^Tc]Tc-MDP SPECT/CT imaging.

**Materials and methods:**

Twenty rats were randomly assigned to a superior cervical ganglionectomy (SCGx) group (n = 10) or a sham-operated control group (n = 10). Circular cranial fractures were surgically created in both groups. Micro SPECT/CT imaging was performed at 3, 6, and 9 weeks postoperatively to assess bone mineral density (BMD), bone volume/tissue volume (BV/TV), and bone volume (BV). In a separate experiment, 12 additional rats underwent either bilateral lumbar sympathectomy (n = 6) or sham operation (n = 6). At 9 weeks, [^99m^Tc]Tc-MDP biodistribution in harvested bone tissues was measured. Immunohistochemical staining for tyrosine hydroxylase (TH) and Ki67 was used to evaluate sympathetic innervation and cell proliferation in craniums, while immunofluorescence co-labeling for Ki67 and osteopontin (OPN) identified proliferating osteoblasts. *In vitro*, MC3T3-E1 osteoblasts were treated with norepinephrine (NE) or control medium for 24 hours. Cell proliferation was assessed using EdU staining. Additionally, sympathetic neurons isolated from neonatal rats were co-cultured with MC3T3-E1 cells in Transwell systems, and mineralization and alkaline phosphatase (ALP) activity were evaluated.

**Results:**

Successful SCGx was confirmed by signs of Horner’s syndrome. SCGx rats exhibited significantly higher [^99m^Tc]Tc-MDP uptake and increased BMD, BV/TV, and BV in peri-fracture regions at all time points (*p* < 0.0001). Lumbar sympathectomy increased tracer uptake in femurs, tibias, lumbar vertebrae, and sacra (*p* < 0.01), but not in cervical or thoracic vertebrae. TH expression decreased, while Ki67 and OPN levels increased in SCGx craniums. NE suppressed MC3T3-E1 proliferation (*p* < 0.0001), and co-culture with sympathetic neurons reduced mineralization and ALP activity (both *p* < 0.0001).

**Conclusion:**

Sympathectomy can enhance osteoblast prolifeation and augment bone formation, which can be effectively assessed and quantified using [^99m^Tc]Tc-MDP SPECT/CT imaging.

## 1 Introduction

Numerous studies have demonstrated that the sympathetic nervous system (SNS) exerts significant regulatory effects on bone formation (1, 2). However, the role of SNS in bone remodeling remains controversial. While some studies suggest that SNS activation inhibits bone formation via β2-adrenergic receptors, others propose a pro-osteogenic effect under specific physiological conditions (3). Most existing research has focused on indirect hypothalamic-mediated central nervous system (CNS) regulation of bone metabolism, whereas direct sympathetic regulation of osteogenesis remains poorly characterized. Bone formation is a critical phase of fracture healing, a complex process involving sequential bone resorption and subsequent deposition to replace damaged tissue (4). Despite its clinical relevance, the interplay between SNS and fracture healing—particularly the balance between osteoblastic bone formation and osteoclastic resorption—has not been thoroughly investigated.

^99m^Tc-methylene diphosphonate ([^99m^Tc]Tc-MDP), a radiopharmaceutical agent widely used in skeletal scintigraphy, provides a functional assessment of bone metabolism by targeting sites of active osteoblastic differentiation and mineralization (5–7). Unlike conventional X-ray imaging, which primarily visualizes structural callus formation, [^99m^Tc]Tc-MDP scintigraphy directly correlates with dynamic metabolic activity, offering superior sensitivity for detecting subtle changes in bone turnover (7, 8). Furthermore, the high sensitivity and specificity of [^99m^Tc]Tc-MDP make it powerful for detecting subtle changes in bone metabolism (9), which can not be analyzed by Micro-CT. These attributes make [^99m^Tc]Tc-MDP bone scan the only tool availabe for evaluating the activity of bone formation *in vivo* following sympathetic denervation.

This study aims to investigate the direct effects of SNS ablation on bone metabolism. For this purpose, superior cervical ganglionectomy (SCGx) was first performed to achieve localized sympathetic denervation in the cranium, following by establishment of a circular cranial fracture in the form of harvesting a circular bone flap and placing it back *in situ*. Afterwards, the [^99m^Tc]Tc-MDP SPECT/CT imaging was employed to chronologically monitor fracture repair (10, 11). Then, the lumbar sympathectomy was performed in rats, and the [^99m^Tc]Tc-MDP SPECT imaging was adopted to evaluate the biodistribution of [^99m^Tc]Tc-MDP in different skeletal sites. Both sympathectomy models revealed significantly enhanced the uptake of [^99m^Tc]Tc-MDP compared to controls. Our findings provide novel insights into the regulatory role of SNS in osteogenesis and validate the utility of [^99m^Tc]Tc-MDP as a functional biomarker for bone metabolism assessment.

## 2 Materials and methods

### 2.1 Animals

20 female Sprague-Dawley rats weighing 305 ± 30 g were used for the study. The rats were singly housed in the same temperature and humidity-controlled room on a 12 h:12 h light-dark cycle. Food and water were available ad libitum. All procedures followed the guidelines established by the National Institutes of Health for the care and use of laboratory animals and received approval from the Experimental Animal Ethics Committee of Fujian Medical University.

### 2.2 Animal models

#### 2.2.1 Performing superior cervical ganglionectomy followed by establishment of a circular cranial fracture

**Figure 1:**
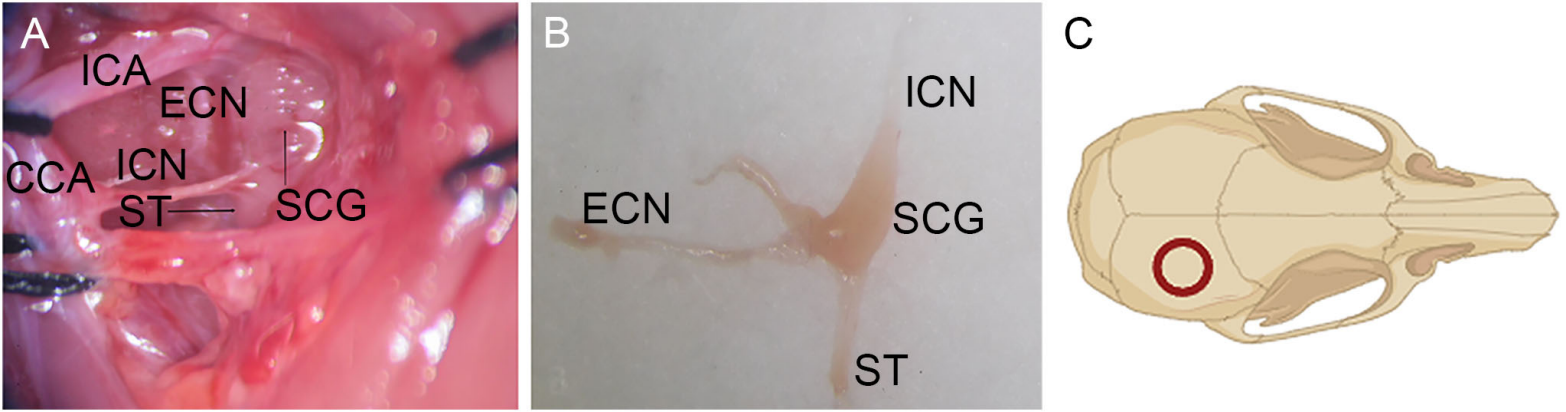
Surgical exposure of SCG. **(A)** The SCG could be consistently identified behind the bifurcation of the common carotid artery into the external and internal carotid arteries. **(B)** The resected SCG and its branches. **(C)** The location of the bone flap harvested on the cranium. ICA, Internal Carotid Artery; ECN, External Cervical Nerve; ICN, Internal Cervical Nerve; ST, Sympathetic Trunk; CCA, Common Carotid Artery.

The rats were randomly divided into two groups. The SCGx (superior cervical ganglionectomy) group (n = 10) underwent right superior cervical ganglionectomy. The rats were anesthetized with pentobarbital sodium (50 mg/kg). The depth of anesthesia was determined by the absence of pedal reflex to a toe pinch, and additional doses of anesthesia were administered as needed. The ventral neck region was shaved and swabbed with 75% alcohol. The subcutaneous tissue and muscles were incised layer by layer to expose the right sternocleidomastoid muscle. which was retracted to expose the carotid triangle to identify the superior cervical ganglion (SCG) (**Figure 1**). The SCG was gently removed, and its branches along with part of the cervical sympathetic trunk were excised, ensuring complete removal. The control group (n = 10) underwent exposure of the carotid triangle without resecting the SCG.

Two days later, the rats were anesthetized again, the skin overlying the scalp was shaved, sterilized, and incised longitudinally (approximately 3 cm in length). After removal of the periosteum, a bone flap with a diameter of 5 mm was harvested using a 5 mm diameter trephine (**Figure 1**). The circular bone flap site was continuously flushed with sterile saline to remove the bone debris and minimize heat produced during drilling. Afterwards, the bone flap was placed back *in situ*. The wound was closed by stitching the scalp.

#### 2.2.2 Surgical procedure for lumbar sympathectomy

Another 12 rats were randomly divided into two groups. The lumbar sympathectomy group (n = 6) underwent lumbar sympathectomy, and the control group (n = 6) underwent a sham operation. In the lumbar sympathectomy group, bilateral lumbar sympathetic trunks were removed following the procedures as previously described (12). Briefly, a longitudinal incision from the xiphoid process to the pubic symphysis was first made along the abdominal skin and the linea alba to open the abdominal cavity. Upon entering the abdomen, the intestine, spleen, liver, and stomach were retracted to the right after freeing the posterior peritoneal attachments. A blunt dissection was carried out to peel off the peritoneal covering of the posterior abdominal wall to explore deeper into the gutter between the psoas major muscles lying over the lumbar vertebrae. The usual four pairs of sympathetic ganglia were then removed under a stereomicroscope at 2× magnification. The rats that underwent lumbar sympathectomy were relatively weak after surgery, and they were kept at a temperature of 28°C until they regained mobility.

### 2.3 Radiotracer preparation

To prepare the [^99m^Tc]Tc-MDP, ^99m^Tc, as [^99m^Tc]NaTcO_4_, freshly prepared from a ^99^Mo/^99m^Tc generator, was added to a kit of MDP. In detail, a vial of Methylene diphosphonate and Stannous Chloride for Injection was obtained. Prior to use, under aseptic conditions, 4-6 mL of [^99m^Tc]NaTcO_4_, injection solution was drawn into the vial according to its radioactive concentration and shaken thoroughly to dissolve the lyophilized powder. After standing for 5 minutes, [^99m^Tc]Tc-MDP injection solution was prepared. The radiochemical yield was measured using radio-TLC. [^99m^Tc]Tc-MDP were diluted to the appropriate concentration using sterile saline before injection into the animals.

### 2.4 Micro SPECT/CT imaging and analysis

After the abovementioned procedures, all rats were subjected to Micro SPECT/CT imaging. All Rats were injected with [^99m^Tc]Tc-MDP (111 MBq, 500 μL) via the tail vein. Subsequently, the rats were placed on the imaging bed under 2% (v/v) isoflurane anesthesia and imaged under continuous 1.5% (v/v) isoflurane anesthesia using a micro SPECT/CT system (Mediso Medical Solutions, Inc., Hungary) (13). CT images were acquired with parameters of 50 kV source voltage, 0.61 mA current, and 300 ms exposure time. Pinhole SPECT images were obtained with the energy peak 140 keV, window width 20%, and a frame rate of 18 frames per second.

**Figure 2:**
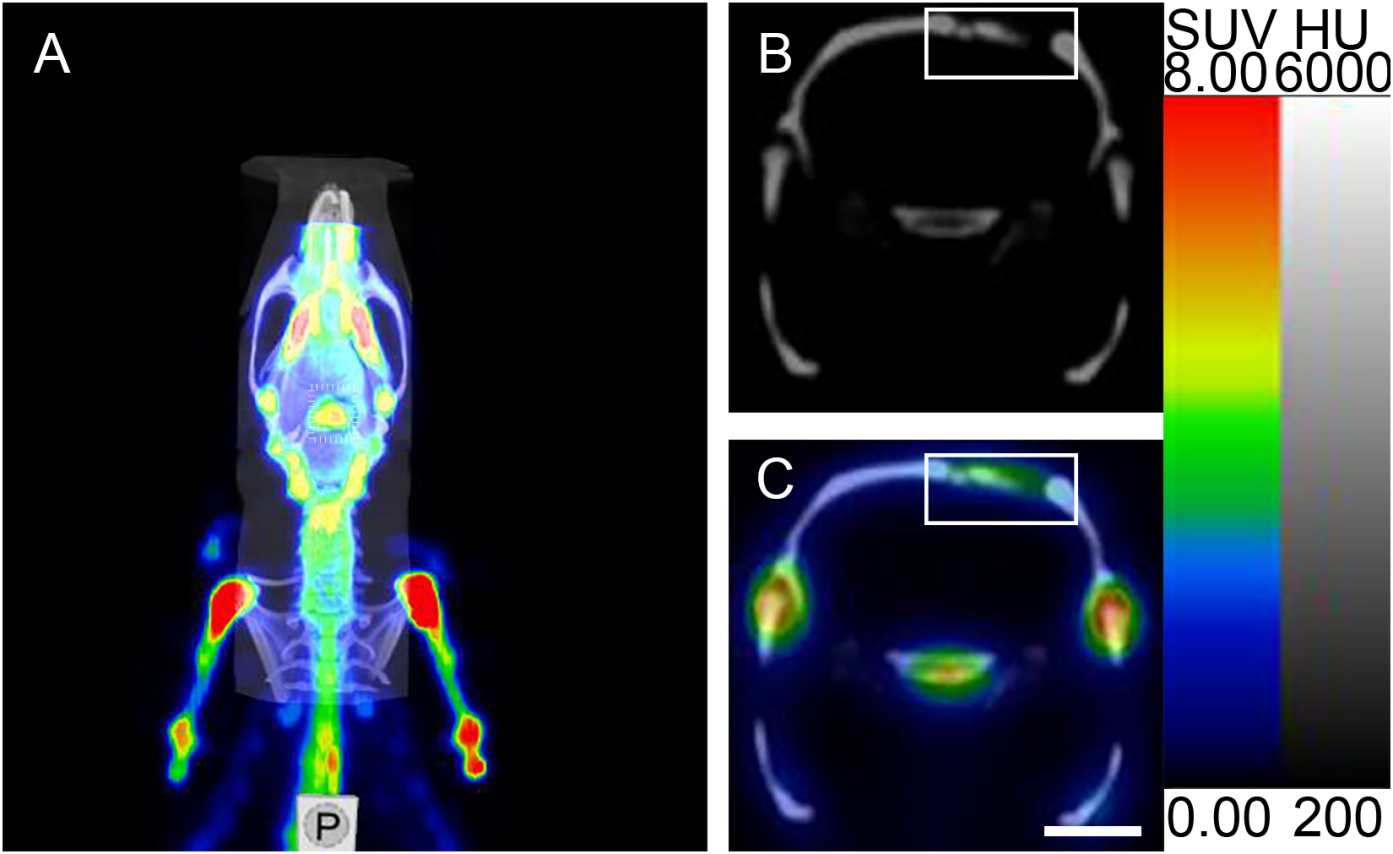
Micro SPECT/CT imaging analysis of ROI. **(A)** Micro SPECT/CT fusion image. **(B, C)** Micro-CT transverse slice and Micro SPECT/CT fusion transverse slice showing [^99m^Tc]Tc-MDP in the area surrounding the cranial fracture(the rectangular region represents the cranial fracture and the area of [^99m^Tc]Tc-MDP uptake). Scale bar: 5 mm.

Three-dimensional SPECT data were acquired and reconstructed using CT-based attenuation correction and an iterative reconstruction algorithm. The CT component was reconstructed using filtered back-projection. Tissue-associated radioactivity was expressed as SUV. SPECT scans were conducted 60 minutes post-injection (n = 5) with CT images acquired simultaneously. SPECT/CT fusion images were analyzed by Nucline NanoScan software (InterView FUSION, Mediso Medical Solutions, Inc., Hungary). For the SCGx group (n = 10) and the control group (n = 10), micro SPECT/CT imaging were performed at 3, 6, 9 weeks, postoperatively. The region of interest (ROI) over the cranium was selected on the transverse slices of the micro SPECT/CT fusion images (**Figure 2**). The reconstructed images were post-processed with a 3D Gaussian filter and displayed with a slice thickness consistent with SPECT scanning. Bone mineral density (BMD), bone volume (BV), and the ratio of bone volume to tissue volume (BV/TV) were quantified from a defined cylindrical volume of interest (VOI) using CTAn software. A VOI (diameter: 5.4 mm, thickness: 1.2 mm) was selected for analysis. BMD was calculated by converting X-ray attenuation coefficients within the VOI into density values using calibration with hydroxyapatite phantoms. BV and BV/TV were obtained by applying appropriate thresholding to binarize the images, allowing for segmentation of bone tissue, and quantifying the corresponding volume (14, 15).

### 2.5 Biodistribution of radioactivity in different skeletal sites *in vivo*

For the lumbar sympathectomy group (n = 6) and the control group (n = 6), the rats were injected with [^99m^Tc]Tc-MDP (7.4 MBq, 100 μL) via the tail vein. After injection, the puncture site was pressed with an alcohol swab. The rats were euthanized at 1 h post-injection, and the cervical vertebrae, thoracic vertebrae, lumbar vertebrae, sacrum, bilateral femurs, and bilateral tibias were harvested, weighed, and quantified for radioactivity in the γ counter (PerkinElmer Wizard2 2480, USA). The uptake value was given as the percent uptake of the injected dose per gram (% ID/g). Tissue samples were trimmed, and all radioactivity measurements were corrected for decay. The percentage of injected dose per gram of tissue (%ID/g) was calculated using the following formula: %ID/g = (tissue radioactivity counts)/(injected radioactivity counts × tissue mass) * 100%.

### 2.6 Histological analysis

Histological analysis was performed to further evaluate the healing of fractures. Cranium samples were decalcified in 10% EDTA for 4 weeks with daily changes. Paraffin-embedded tissues with 5 μm thickness were dewaxed and rehydrated. Ki67 immunohistochemical staining (Servicebio, China, GB111141), osteopontin (OPN, GB11500, Servicebio, China), tyrosine hydroxylase (TH, Chemicon, China, Ab152) and Ki67 immunofluorescence staining, were performed on the tissue sections to evaluate bone repair according to the manufacturer’s protocols (16, 17). Images were captured by a Nikon microscope and a fluorescence microscope (Leica, DMIL LED).

### 2.7 Osteoblasts culture with Norepinephrine and EdU assay

MC3T3-E1 osteoblasts (Procell, China, CL-0378) were cultured in α-Minimum Essential Medium (α-MEM, Procell, China, #PM150410) supplemented with 10% fetal bovine serum and 1% penicillin/streptomycin (Servicebio, China, G4003-100ML) in a humidified incubator with 5% CO_2_ at 37°C. MC3T3-E1 cells were seeded in a 24-well plate (5 × 10^4^ cells/well) and divided into two groups: the control group and the norepinephrine (NE) group. Since the primary neurotransmitter secreted by the SNS is NE, we used a culture medium supplemented with NE to simulate the cellular environment co-cultured with sympathetic neurons. The cells were incubated in medium with NE at a concentration of 100 μM (MCE, HY-13715) according to a previous report (18). The NE group was incubated in medium containing NE for 24 h, whereas The control group was incubated without the addition of drugs.

The proliferation of cells was detected using a 5-ethynyl-2′-deoxyuridine (EdU) cell proliferation assay according to the manufacturer’s instructions (19). Briefly, cells were incubated at 37°C with 10 μmol/L EdU (Beyotime, China, C0071S) for 7 h, fixed in 4% paraformaldehyde solution (Beyotime, China, P0099-3L) at room temperature for 15 min, permeabilized with 0.5% Triton X-100 (Beyotime, China, ST795) at room temperature for 15 min, and then incubated with the click-reaction reagent for 30 min at room temperature in the dark. The nucleus was counterstained with Hoechst 33342 (1:400, Beyotime, China, C1025) for 5 min at room temperature. Three random fields of each sample were acquired with a 20× objective lens using a Nikon Eclipse-Ti-S fluorescence microscope. The ratio of EdU-positive cells to total Hoechst 33342-positive cells was calculated. for each experiment. Three independent experiments were performed.

### 2.8 Isolation and primary culture of sympathetic neurons and co-culture with osteoblasts

SCGs were harvested under a dissection microscope from newborn rat pups (postnatal day 0 or day 1) and placed into cold serum-free medium (Beyotime, China, C0350-50ml). The attached extraneous tissue was carefully removed. The cleaned SCG was first digested with collagenase (Servicebio, China, GC305015-100mg) at a concentration of 1 mg/mL for 30 minutes and then treated with trypsin (Gibco, China, 15050065) at a concentration of 0.25% for an addtional 30 minutes at 37°C. After incubation, complete medium was added to neutralize the trypsin, and the solution was centrifuged at 1200 rpm for 2 minutes. The supernatant was subsequently discarded. Cells were further dissociated in 2 mL of cold serum-free Neurobasal by gently triturating the ganglia with a fire-polished glass pasture pipette until no visible clumps were left and the solution became cloudy. Finally, cells were seeded onto Transwell inserts (Beyotime, Cnina, FTW070-12Ins) pre-coated overnight with poly-D-lysine (Gibco, China, A3890401), laminin (Gibco, China, 23017015), and rat tail collagen (Procell, China, PB180635). The cells were cultured in DMEM (Meilunbio, China, MA0212) supplemented with 10% fetal bovine serum, 1% penicillin/streptomycin, and 2% B-27 (Gibco, China, A3582801).

For co-culture experiments, 24-well Transwell cell culture chambers (Beyotime, China, FTW070-12Ins) with 0.4 μm pores were used. This pore size permitted the diffusion of soluble factors but prevented cell migration and direct cell contact. MC3T3-E1 osteoblasts were co-cultured with sympathetic neurons, while a monoculture of MC3T3-E1 cells was established in parallel as a control. Co-culture was initiated five days after plating sympathetic neurons, with a neuronal seeding density of 5×10^4^ cells/well. The seeding density of osteoblasts was adjusted according to the experimental requirements. The neurons and osteoblasts shared the same medium, which was a 1:1 mixture of their respective culture media.

### 2.9 Mineralization assay Alizarin Red S staining

MC3T3-E1 cells were seeded in 24-well plates at a density of 1 × 10^5^ cells/well. Cells were divided into two groups: co-culture group, and control group. After the cell attachment, 50 μg/mL ascorbic acid (Beyotime, China, ST1434) and 10 mM β-glycerophosphate (Macklin, China, G799605) were added to the culture medium of both groups, and the solution was replaced every 3 days. After 14 days of incubation, the original culture medium was discarded, and the cells were gently washed twice with PBS. The cells were then fixed with 4% paraformaldehyde for 20 minutes, after which the fixative was discarded and the cells were washed again with PBS twice. Mineralized matrix formation was assessed by Alizarin Red S staining. The cells were stained with Alizarin Red S (Beyotime, China, C0148S) for 30 minutes, then washed with PBS and air-dried. Mineralization nodules were scanned and photographed under a microscope. To quantify matrix mineralization, 10% cetylpyridinium chloride was added to each well and incubated for 1h to dissolve and release the calcium-bound alizarin red. The absorbance of the solution was measured at 562 nm using a spectrophotometer (Biotek Epoch, USA).

### 2.10 Alkaline phosphatase activity assay

Alkaline phosphatase (ALP) is a well-known early marker for osteoblast differentiation. The ALP activity was determined spectrophotometrically. MC3T3-E1 cells were seeded in 24-well plates at a density of 1 × 10^5^ cells/well and divided into two groups: co-culture group, and control group. After the cells adhered to the wall, the culture medium was changed and added with a concentration of 10 mM sodium β-glycerophosphate and 50 μg/mL ascorbic acid. After 7 days of incubation, ALP activity was examined using the ALP assay kit (Beyotime, China, P0321S) according to the manufacturer’s instructions.

### 2.11 Statistical analyses

All data are presented as the Mean ± SD. Statistical analyses were performed using SPSS version 16.0 (Statistical Package for Social Sciences, Chicago, IL). Statistical significance was assessed using independent samples *t* tests. A confidence level of 95% (*p* < 0.05) was considered statistically significant.

## 3 Results

### 3.1 Successful sympathectomy evidenced by Horner’s syndrome and reduced TH expression

**Figure 3:**
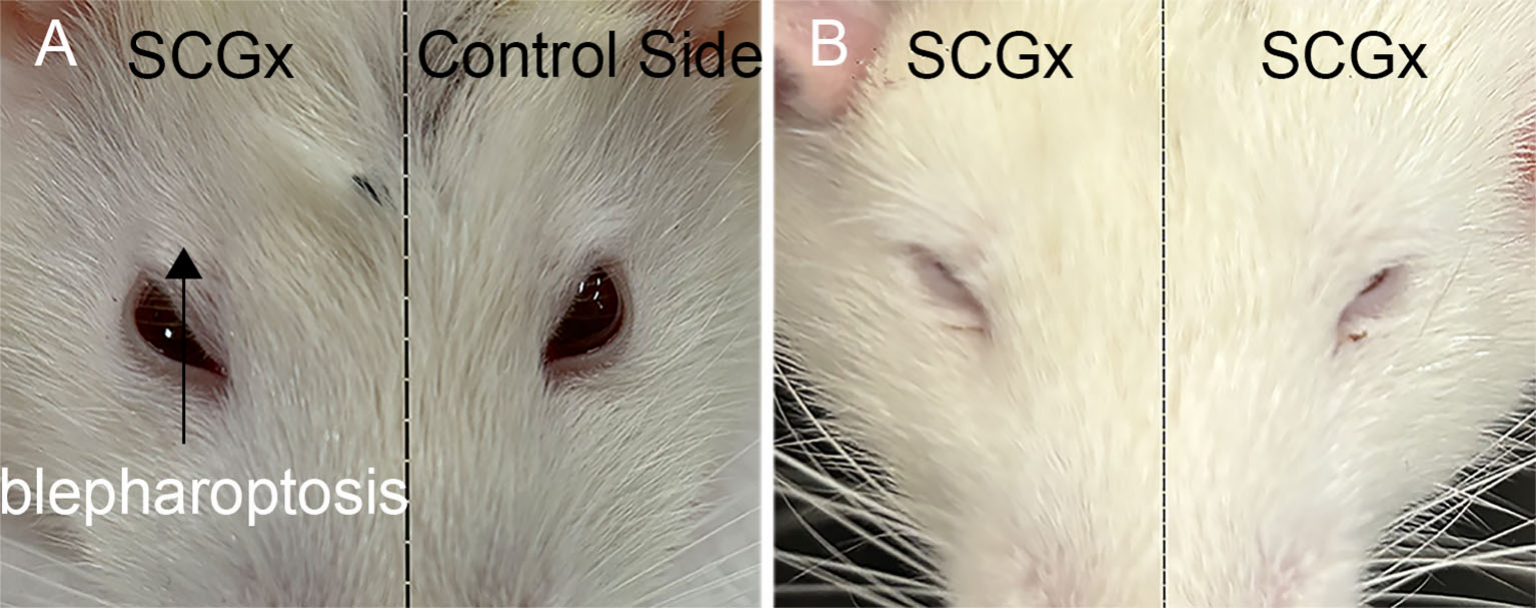
Ptosis characteristic of Horner’s syndrome following right **(A)** and bilateral **(B)** SCGx in rats.

**Figure 4:**
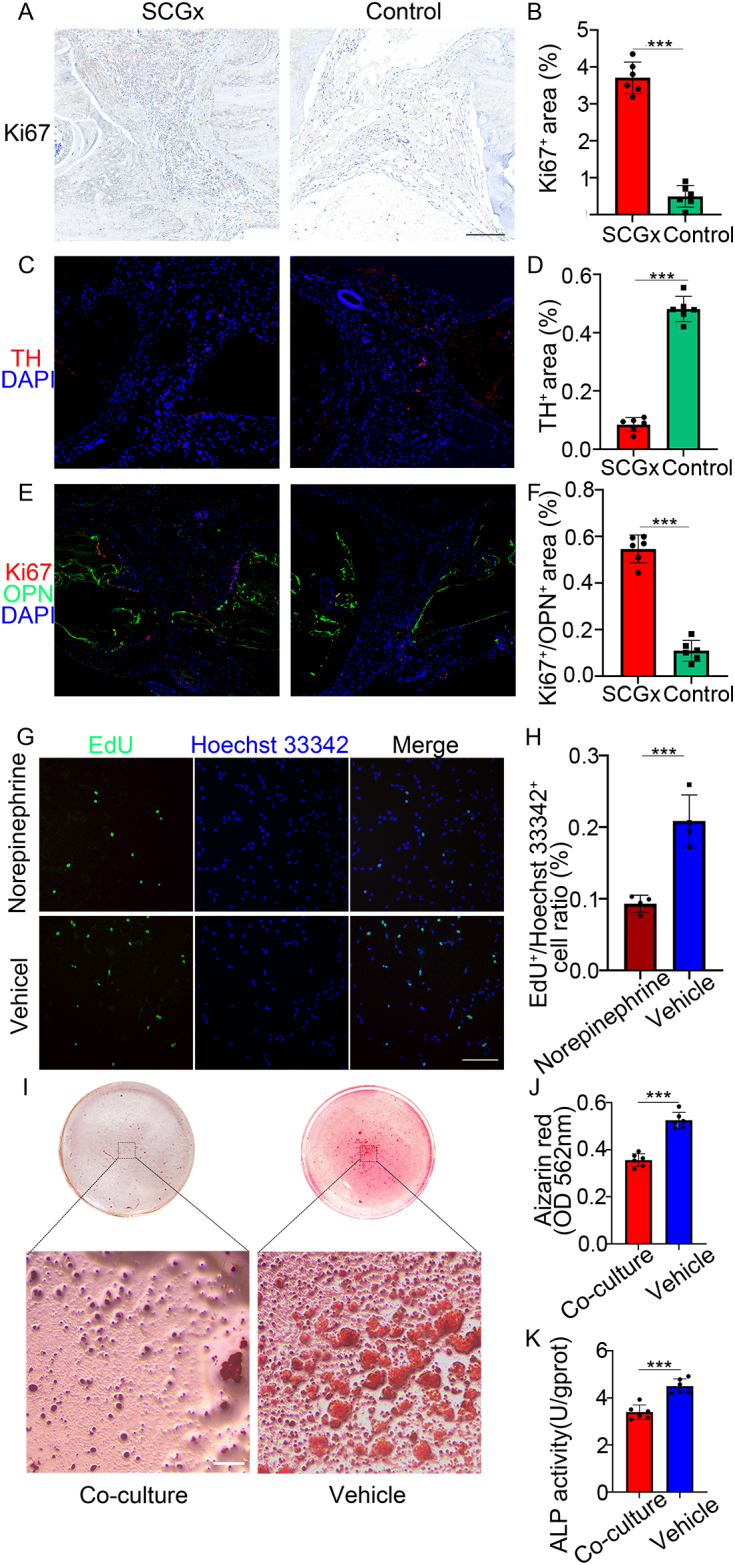
**(A)** Representative images of immunohistochemistry staining of Ki67 (red), scale bar: 5 mm. **(B)** Quantification of the percentage of positive area of Ki67 immunohistochemical staining. **(C)** Representative images of immunofluorescence staining of TH (red), and nuclear counterstaining of DAPI (blue). **(D)** Quantification of the percentage of positive area of TH immunofluorescence staining. **(E)** Representative images of co-labeling immunofluorescence staining of Ki67 (red), OPN (green), and nuclear counterstaining of DAPI (blue). **(F)** Quantification of the percentage of positive area of Ki67 immunofluorescence staining. **(G)** MC3T3-E1 cells were cultured with or without NE (100 μM) for 24 h, followed by EdU incorporation assay. The proliferating cells were labeled with EdU (green), nuclei were stained blue with Hoechst 33342 (blue). Scale bar: 0.2 mm. **(H)** Statistical analysis of EdU-positive/Hoechst 33342-positive cell ratio in two groups. **(I)** Representative images of Alizarin Red S staining showing mineralization in co-culture versus monoculture. Scale bar: 0.25 mm. **(J)** Quantification of alizarin red absorbance at 562 nm. **(K)** Quantification of ALP activity. ***p < 0.0001.

One week after SCGx in the right side, Horner’s syndrome could be observed in the right eye, indicating a successful sympathectomy (**Figure 3**). Consistent with this functional evidence, the SCGx group had a minor expression of TH, whereas the control group exhibited higher levels of TH, with a TH-positive area localized in the periosteal region (**Figure 4**). The TH-positive area percentage was significantly reduced in the SCGx group (0.09 ± 0.02%) compared to the control group (0.48 ± 0.04%, *p* < 0.0001).

### 3.2 Increased [^99m^Tc]Tc-MDP in the areas surrounding the fracture following SCGx

The radiochemical purity of the [^99m^Tc]Tc-MDP injection solution was determined to be greater than 90% prior to use (**Supplementary Figure 1**).

**Figure 5:**
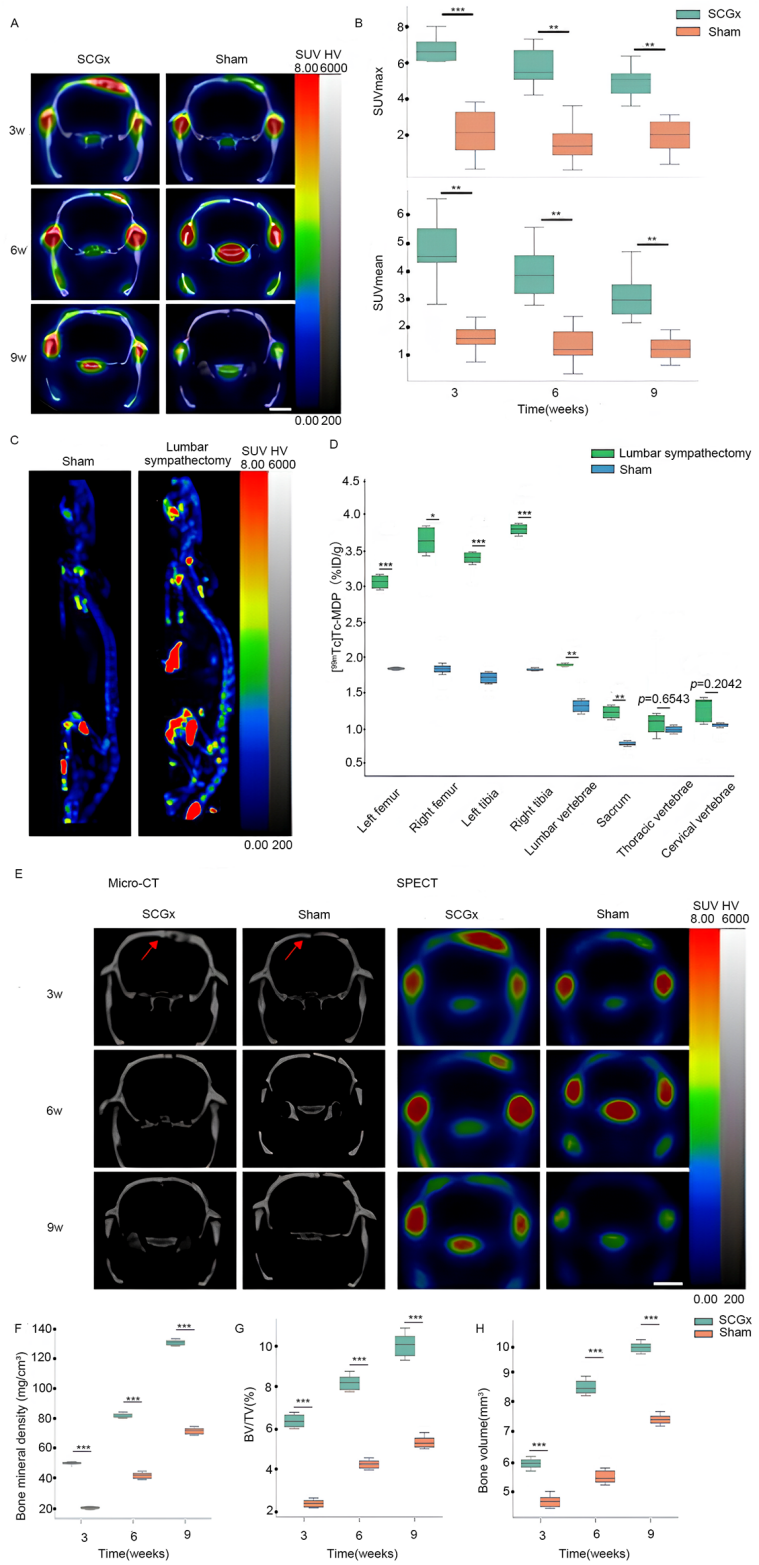
**(A)** Representative micro SPECT/CT fusion images. The color scale represents the level of tissue-associated radioactivity, with red representing the highest uptake, scale bar: 5 mm. **(B)** The uptake of [^99m^Tc]Tc-MDP after SCGx. **(C)** Representative images of whole-body SPECT scans. **(D)** [^99m^Tc]Tc-MDP biodistribution in different skeletal sites. **(E)** Representative micro-CT and SPECT images. The color scale represents the level of tissue-associated radioactivity, with red representing the highest uptake, scale bar: 5 mm. Red arrows indicate the sagittal suture. **(F–H)** Summarized data showing the micro-architectural parameters of the newly formed bone tissue by analyzing micro-CT images using image analysis software. BMD (mg/cm³), BV/TV (%) and BV (mm³). ***p < 0.0001. signifies italic p < 0.01.

Three weeks after SCGx, micro SPECT/CT fusion images showed markedly higher tissue radioactivity in the areas surrounding the cranial fracture of the SCGx group (n = 10) compared to the control group (n = 10). Nine weeks after SCGx, the control group exhibited consistently low tissue radioactivity in the areas surrounding the fracture. Quantitative analysis revealed [^99m^Tc]Tc-MDP was significantly higher within the area surrounding the fracture of the SCGx group compared to the control group at 3 weeks, 6 weeks, and 9 weeks (all *p* < 0.0001) after SCGx, respectively (**Figure 5A**). Detailed data were listed in **Table 1**.

**Table 1:** The uptake of [^99m^Tc]Tc-MDP after SCGx (n = 20).

Time (weeks)	Group	SUVmean	SUVmax
3	SCGx	4.24 ± 1.17	6.87 ± 1.07
	Sham	1.61 ± 0.36	2.41 ± 1.00
6	SCGx	3.59 ± 0.92	5.67 ± 1.08
	Sham	1.38 ± 0.29	1.65 ± 0.48
9	SCGx	3.18 ± 1.37	4.87 ± 1.26
	Sham	1.22 ± 0.55	1.87 ± 1.06

Three weeks after SCGx, micro-CT transverse slices showed bone resorption and blurred shadows surrounding the fracture site in the SCGx group (n = 10), whereas no significant changes were observed in the control group (n = 10). Six weeks after SCGx, bone fusion was observed on the side of the fracture line distant from the sagittal suture in the SCGx group, while the fracture line near the sagittal suture appeared blunt. Nine weeks after SCGx, bone fusion was observed near the sagittal suture in the SCGx group, while the control group showed no significant changes in bone formation or resorption. Quantitative micro-CT analysis revealed that the bone histomorphometric parameters (BMD, BV/TV, and BV) were significantly higher in the SCGx group than in the control group at 3, 6, and 9 weeks after SCGx (**Figures 5E–H**, all *p* < 0.0001). Detailed data were listed in **Table 2**.

**Table 2:** The bone histomorphometric parameters after SCGx (n = 20).

	SCGx			Sham		
Time (weeks)	BMD (mg/cm³)	BV/TV (%)	BV (mm³)	BMD (mg/cm³)	BV/TV (%)	BV (mm³)
3	50.13 ± 0.93	6.52 ± 0.31	5.99 ± 0.18	20.10 ± 0.90	2.51 ± 0.23	4.64 ± 0.22
6	81.62 ± 1.77	8.18 ± 0.46	8.58 ± 0.31	40.93 ± 2.35	4.35 ± 0.21	5.58 ± 0.23
9	130.67 ± 1.46	9.88 ± 0.51	9.57 ± 0.19	71.00 ± 2.24	5.50 ± 0.30	7.40 ± 0.16

### 3.3 Lumbar sympathectomy increases [^99m^Tc]Tc-MDP uptake in lower limb bones, lumbar vertebrae and sacrum

Nine weeks after surgery, the lumbar sympathectomy group (n = 6) exhibited significantly higher radioactivity levels compared to the control group (n = 6) in the left femur, right femur, left tibia, right tibia, lumbar vertebrae, and sacrum (all *p* < 0.01). However, no significant differences were observed in the cervical vertebrae (*p* = 0.2042) and thoracic vertebrae (*p* = 0.6543) between the two groups (**Figure 5C**). Detailed data were listed in **Table 3**.

**Table 3:** [^99m^Tc]Tc-MDP biodistribution in different skeletal sites (n = 12).

Bone tissues	Lumbar sympathectomy (%ID/g)	Sham (%ID/g)	*p-*value
Left femur	3.07 ± 0.34	1.85 ± 0.02	< 0.0001
Right femur	3.59 ± 0.50	1.85 ± 0.22	< 0.01
Left tibia	3.46 ± 0.25	1.63 ± 0.24	< 0.0001
Right tibia	3.84 ± 0.25	1.81 ± 0.09	< 0.0001
Lumbar vertebrae	1.89 ± 0.08	1.35 ± 0.32	< 0.001
Sacrum	1.26 ± 0.34	0.74 ± 0.12	< 0.001
Cervical vertebrae	1.44 ± 0.38	1.12 ± 0.08	0.2042
Thoracic vertebrae	1.16 ± 0.39	1.02 ± 0.17	0.6543

### 3.3 SCGx enhances osteogenic cell proliferation

Histological examination of cranium sections revealed notable differences between the SCGx and control groups (**Figures 5A–F**). Immunohistochemical staining for Ki67 demonstrated a significantly higher proliferative activity in the SCGx group, with Ki67-positive areas occupying 3.78 ± 0.90% of the fracture zone compared to 0.44 ± 0.50% in the control group (*p* < 0.0001). immunofluorescence Co-labeling further revealed that Ki67/OPN double-positive area accounted for 0.63 ± 0.11% in the SCGx group, compared to 0.11 ± 0.04% in the control group (*p* < 0.0001), suggesting an increased population of proliferating osteogenic cells. Morphologically, the SCGx group exhibited irregular, actively remodeling fracture margins contrasting with the sharp, quiescent fracture lines in the control group.

### 3.4 Inhibition of proliferation of MC3T3-E1 osteoblasts by NE

EdU incorporation assays demonstrated a reduced proportion of EdU-positive cells in the NE group compared to the control group (**Figure 4**), indicating NE significantly suppressed the proliferation of MC3T3-E1 osteoblasts. After 24 h of treatment, the ratio of EdU-positive cells to total Hoechst 33342-positive cells in the NE group was significantly lower at 9.32 ± 1.02% compared to 20.82 ± 3.19% in the control group (**Figure 4**, *p* < 0.0001).

### 3.5 Co-culture of sympathetic neurons inhibits osteoblast mineralization and differentiation

The mineralization assay by Alizarin Red S staining indicated that the mineralization node area and the absorbance of the alizarin red dye in MC3T3-E1 cells were obviously decreased after 14 days of co-culture with sympathetic neurons (**Figures 4I**, *p* < 0.0001). Quantitatively, the alizarin red absorbance at 562 nm was 0.36 ± 0.03 in the co-culture group, significantly lower than 0.53 ± 0.03 in the monoculture control group. Moreover, the ALP activity of MC3T3-E1, an early marker of osteoblast differentiation, was reduced after 7 days of co-culture with sympathetic neurons (3.49 ± 0.34 U/gprot) compared to MC3T3-E1 monoculture (4.48 ± 0.32 U/gprot) (**Figure 4**, *p* < 0.0001).

## 4 Discussion

Most existing studies on regulation of bone metabolism by sympathetic nerves focus on the CNS, particularly the hypothalamic control of sympathetic nerves through endocrine function, which subsequently affects bone metabolism (20, 21). In contrast, this study explores a new perspective by investigating the direct regulation of bone metabolism by sympathetic nerves. The conventional approach to achieve sympathetic denervation primarily involves the use of 6-hydroxydopamine (6-OHDA), which selectively destroys sympathetic nerve terminals by accumulation of the neurotoxin specifically via noradrenergic transporters, causing a depletion of NE and reduced TH activity (22, 23). In contrast, our study establishes a model of localized surgical sympathetic denervation that more closely mimics clinical surgical procedures. Selective removal of the SCG in the neck region avoids potential metabolic disturbances associated with systemic sympathectomy, thereby providing a more precise model to assess the effects of sympathetic denervation on bone metabolism in the cranium.

Although traditional X-ray and micro-CT are widely used in studies of fracture healing, they cannot directly measure bone metabolism during this process. In contrast, [^99m^Tc]Tc-MDP SPECT/CT bone scan enables dynamic evaluation of bone formation. This study combines micro-CT with SPECT, using accurate localization to analyze the level of bone metabolism after sympathectomy, providing a novel method for investigating the complex role played by SNS in bone metabolism. [^99m^Tc]Tc-MDP imaging has great application prospects in assessing bone metabolic activity. This radiotracer exhibits a strong affinity for bone and can specifically bind to the surface of hydroxyapatite crystals through chemisorption. Moreover, it can also associate with organic components of the bone matrix, such as collagen fibers, and be incorporated into mature bone, allowing the radionuclide to accumulate within the bone tissue for imaging. As a sensitive bone imaging agent, the accumulation of [^99m^Tc]Tc-MDP is closely correlated with bone mineralization and osteoblast differentiation (24, 25). Leveraging this advantage, we utilized [^99m^Tc]Tc-MDP to evaluate bone formation in fracture healing. Rats in the SCGx group exhibited significantly higher [^99m^Tc]Tc-MDP in areas surrounding the cranial fracture than the control group from 3 to 9 weeks after sympathetic denervation, with the most significant difference at 3 weeks, postoperatively. This result is consistent with the radioactive decay of [^99m^Tc]Tc-MDP, as its uptake declined over time. A substantial amount of radionuclide was lost to natural decay due to the relatively short half-life of ^99m^Tc, which is approximately 6 hours (26). Furthermore, [^99m^Tc]Tc-MDP distribution in the lumbar sympathectomy group was significantly higher in lumbar vertebrae, sacral vertebrae, bilateral femurs, and bilateral tibias which are regions innervated by the lumbar sympathetic trunk, compared to the control group at 9 weeks, postoperatively. However, no statistically significant differences were observed in the cervical and thoracic vertebrae regions (*p* > 0.05). These findings from distinct surgical procedures indicate that sympathetic denervation can lead to an increase in the uptake of [^99m^Tc]Tc-MDP. What is worth noting is that we did not establish a model of lower limb fractures following lumbar sympathectomy due to technical limitations. Our initial attempt to establish a transverse tibial fracture model with internal fixation, as described by Wu et al. (27), encountered high infection and mortality rates due to Kirschner wire placement difficulties and postoperative wound biting by rats. Additionally, we explored a closed tibial fracture model using a modified three-point bending pliers based on Handool et al. (28). However, due to experimental constraints, we were unable to achieve a standardized tibial fracture model. In contrast, circular cranial fractures offer advantages such as procedural simplicity and low mortality rates, making them a more feasible approach to study the impact of sympathectomy on fracture healing. Therefore, in this study, we only performed cranial fractures post-SCGx to ensure the reproducibility.

Micro-CT analysis of bone morphology revealed that, after sympathectomy, new bone, BMD, BV/TV and BV surrounding the bone flap were higher in the SCGx group compared with the control group. This finding is consistent with the increased [^99m^Tc]Tc-MDP observed in the SCGx group, as shown by SPECT imaging. Histological analysis further supports these results, indicating that sympathetic denervation stimulated cellular proliferation during fracture healing, aligning with both the increased [^99m^Tc]Tc-MDP and enhanced bone formation observed in the SCGx group. Nine weeks after SCGx, it is worth noting that the TH-positive area was still present in small amounts in the SCGx group. We hypothesize that this may be due to partial regeneration of sympathetic nerve fibers. Despite this residual sympathetic activity, the overall trend observed in this study suggests that sympathectomy promotes bone formation. Augmentation of bone formation following sympathectomy as observed in this study can be attributable to two factors. First, surgical sympathetic denervation can result in dilation of arteries, causing increased local blood supply (12), which may contribute to the increased bone formation observed in the area surrounding the cranial fracture. Second, sympathetic nerves may directly regulate bone formation independent of their vascular effects. To substantiate this hypothesis, we conducted *in vitro* experiments by supplementing the culture of the mouse MC3T3-E1 osteoblasts with NE, the principal neurotransmitter released from sympathetic nerves. According to a study by Guo et al. (18), the addition of an appropriate concentration of NE to osteoblast culture medium partially mimics the co-culture of sympathetic nerves and osteoblasts. Compared to the saline control group, NE treatment significantly attenuated cell proliferation, implying increased sympathetic activity inhibits osteoblastic activity. This direct inhibitory effect of NE on osteoblasts provides a plausible explanation for the enchanced bone formation upon sympathetic denervation. In addition, as previous research by Yang et al. (29), we also performed co-culture experiments with sympathetic neurons and MC3T3-E1 osteoblasts to examine the effects of sympathetic denervation on osteoblast differentiation and mineralization *in vitro*. Compared to monoculture, the differentiation and mineralization levels of MC3T3-E1 osteoblasts were significantly decreased in the co-culture group, providing additional evidence that sympathetic neuron activity plays an inhibitory role in osteoblast function.

Our findings confirm the inhibitory effect of SNS on bone formation reported by Elefteriou F. et al. (30). However, the study by Liu. et al. (31) found that increased sympathetic excitation after traumatic brain injury can promote fracture healing, but this study failed to further analyze the effects of other parts of the nervous system besides the SNS after brain trauma, thus having certain limitations. In our study, not only did sympathectomy lead to increased [^99m^Tc]Tc-MDP uptake and enhanced bone formation, but the proliferation of MC3T3-E1 osteoblasts was also inhibited in culture media containing NE. These findings collectively indicate that SNS exerts a direct inhibitory effect on bone formation, an effect independent of central regulation. Therefore, our study provides a complement to the prevailing view that sympathetic regulation of bone metabolism is primarily mediated through the hypothalamus and its downstream endocrine factors. However, a limitation of this study is that only unilateral sympathetic denervation of the neck was performed, as bilateral superior cervical ganglionectomy resulted in high mortality rates in rats. Furthermore, this study has several limitations that warrant attention. First, only unilateral superior cervical ganglionectomy was performed because bilateral procedures resulted in high mortality rates. Second, the long-term effects of this procedure on bone formation remain unclear. Finally, the lack of biomechanical and functional data hinders the interpretation of whether increased bone formation enhances mechanical strength. To resolve these limitations, future studies will incorporate biomechanical testing (e.g., three-point bending and nanoindentation) and functional evaluations (e.g., gait analysis). Additionally, they will systematically examine the potential impact of [^99m^Tc]Tc-MDP administration on bone metabolism itself.

In conclusion, this study is the first to utilize [^99m^Tc]Tc-MDP SPECT/CT imaging as a measure to evaluate neurogenic regulation of bone metabolism. By surgically sympathetic denervation, we demonstrate a promotive effect on bone formation in rats cranial fracture. This represents an important preclinical study elucidating the regulatory role of sympathetic nerves in bone repair.

## Data Availability

The original contributions presented in the study are included in the article/**Supplementary Material**. Further inquiries can be directed to the corresponding authors.
