# Supplementary figures and images for: Augmentation of bone formation by sympathectomy in rats as evaluated by [99mTc]Tc-MDP
Source: Front Endocrinol (Lausanne). 2025 Jul 2;16:1580230. doi: 10.3389/fendo.2025.1580230 (PMC12264536; doi:10.3389/fendo.2025.1580230)

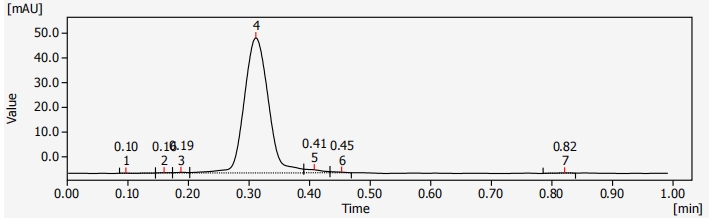

Supplement: Supplementary file 1 [file Image1.png]
